# Metacognitive bias resulting from trade-off between local and global motion signals

**DOI:** 10.1167/jov.23.10.7

**Published:** 2023-09-11

**Authors:** Alan L. F. Lee, Hana Yabuki, Isaac C. L. Lee, Charles C.-F. Or

**Affiliations:** 1 Department of Psychology, Lingnan University, Hong Kong; 2 Division of Psychology, School of Social Sciences, Nanyang Technological University, Singapore; 3 Department of Psychology, Lingnan University, Hong Kong; 4 Division of Psychology, School of Social Sciences, Nanyang Technological University, Singapore

**Keywords:** metacognition, confidence, global and local motion, hierarchical processing, psychophysics

## Abstract

Visual confidence generally depends on performance in targeted perceptual tasks. However, it remains unclear how factors unrelated to performance affect confidence. Given the hierarchical nature of visual processing, both local and global stimulus features can influence confidence, but their strengths of influence remain unknown. To address this question, we independently manipulated the local contrast signals and the global coherence signals in a multiple-aperture motion pattern. The drifting-Gabor elements were individually manipulated to give rise to a coherent global motion percept. In both dichotomous direction-discrimination task (Experiment 1) and analog direction-judgment task (Experiment 2), we found stimulus-dependent biases in metacognition despite matched perceptual performance. Specifically, participants systematically gave higher confidence ratings to an incoherent pattern with clear elements (i.e., strong local but weak global signals) than a coherent pattern with noisy elements (i.e., weak local but strong global signals). We did not find any systematic effects of local/global stimulus features on metacognitive sensitivity. Model comparisons show that variation in local/global signals in the stimulus should be considered a factor influencing confidence, even after controlling for the effects of performance. Our results suggest that the metacognitive system, when generating confidence for a perceptual task, puts more weights on local than global signals.

## Introduction

Visual confidence refers to observers’ judgment about the accuracy of their own response to a visual task (Mamassian, 2016). As confidence judgments involve the observers’ reflection on their perceptual and/or cognitive processes, they are often regarded as the product of metacognition (Fleming, Dolan, & Frith, 2012).

In addition to performance (see Fleming and Lau, 2014 and Maniscalco and Lau, 2012; Maniscalco and Lau, 2014 for discussion on correlation between confidence and performance), there are multiple factors that can influence visual confidence. Such non-performance-related factors may be manipulated or observed to vary where performance is matched across conditions. Some of these factors could be internal and pertain to the observers, such as attention (e.g., Rahnev et al., 2011) and training (e.g., Carpenter et al., 2019). Other factors could be external, which are related to stimulus features (e.g., Boldt, de Gardelle, and Yeung, 2017; Spence, Dux, & Arnold, 2016; Zylberberg, Roelfsema, & Sigman, 2014). In this article, we focus on the external factors.

The effects of changes in external stimulus features on visual confidence remain obscure. In some studies, stimulus features were manipulated systematically on certain dimensions (e.g., de Gardelle & Mamassian, 2015; Barthelme & Mamassian, 2010). Confidence was found to closely track performance but did not show any systematic change with the manipulated dimensions. Other studies found systematic and predictable changes in confidence after manipulations of a specific stimulus feature despite matched performance, especially in studies that vary the stimulus feature across multiple elements in a stimulus (Boldt et al., 2017; Spence et al., 2016; Zylberberg et al. 2014). Recently, it has been proposed that the metacognitive system could use stimulus features as heuristics when computing visual confidence (Bertana, Chetverikov, van Bergen, Ling, & Jehee, 2021). Notably, understanding the relationship between confidence and stimulus features can be complicated by the fact that visual stimuli contain multiple dimensions of features, which can give conflicting signals along the hierarchy of visual processing.

In low-level, early stages of visual processing (e.g., V1), responses of the orientation-selective units with small and localized receptive fields on specific locations increase with stimulus contrast (e.g., Tolhurst, Movhson, & Thompson, 1981; Sclar & Freeman, 1982). Reducing luminance-contrast signals (e.g., by introducing contrast noise on gratings [e.g., Rausch & Zehetleitner, 2016; Rausch, Hellmann, & Zehetleitner, 2018]) has recently been found to affect both confidence and visibility ratings. The results may be explained by theorizing that introducing uncertainty with low-level contrast noise affects performance, which then affects confidence.

In high-level, later stages of visual processing (e.g., middle temporal (MT) for motion perception), global uncertainty can be manipulated by changing the variability along a stimulus-feature dimension (e.g., orientation) across local elements (e.g., gratings). Higher variability across local elements would lead to greater uncertainty in the integration of local signals to form a global percept. Consider the global motion in a random-dot kinematogram: Each dot is a local element that can be set to move in any direction. When all dots move in the same direction, they will be coherent in representing the same global motion direction. However, when the dots differ substantially in their local motion directions, they will be incoherent in representing the global motion direction, leading to greater uncertainty at the global level. Neural responses of direction-selective units at the global level of motion processing (e.g., MT) increase with the coherence of a random-dot kinematogram (Newsome, Britten, & Movshon, 1989). In the human psychophysics literature, global coherence is also an important factor for perceiving global motion direction (e.g., Mingolla, Todd, & Farley Norman, 1992; Ross, Badcock, & Hayes, 2000; Burr, Morrone, & Vaina, 1998).

If one views the visual system from this hierarchical perspective, it becomes possible that changes in local and global stimulus features affect confidence separately and to different extents. Studying this is important for understanding visual metacognition because it remains unclear (1) what information the metacognitive system takes as input and (2) how such information is used in generating confidence judgments. Clarifying these relationships helps researchers develop falsifiable and detailed computational models for visual metacognition, which has been regarded as one of the long-term goals for the field (Rahnev et al., 2022). Specifically, because metacognition and confidence are often associated with later stages of processing, such as the prefrontal cortex and parietal areas (e.g., Bang & Fleming, 2018; Kiani & Shadlen, 2009; and reviewed in Meyniel, Sigman, & Mainen, 2015), it would be interesting to investigate the extent to which local and global signals work together in influencing visual confidence.

**Figure 1.**
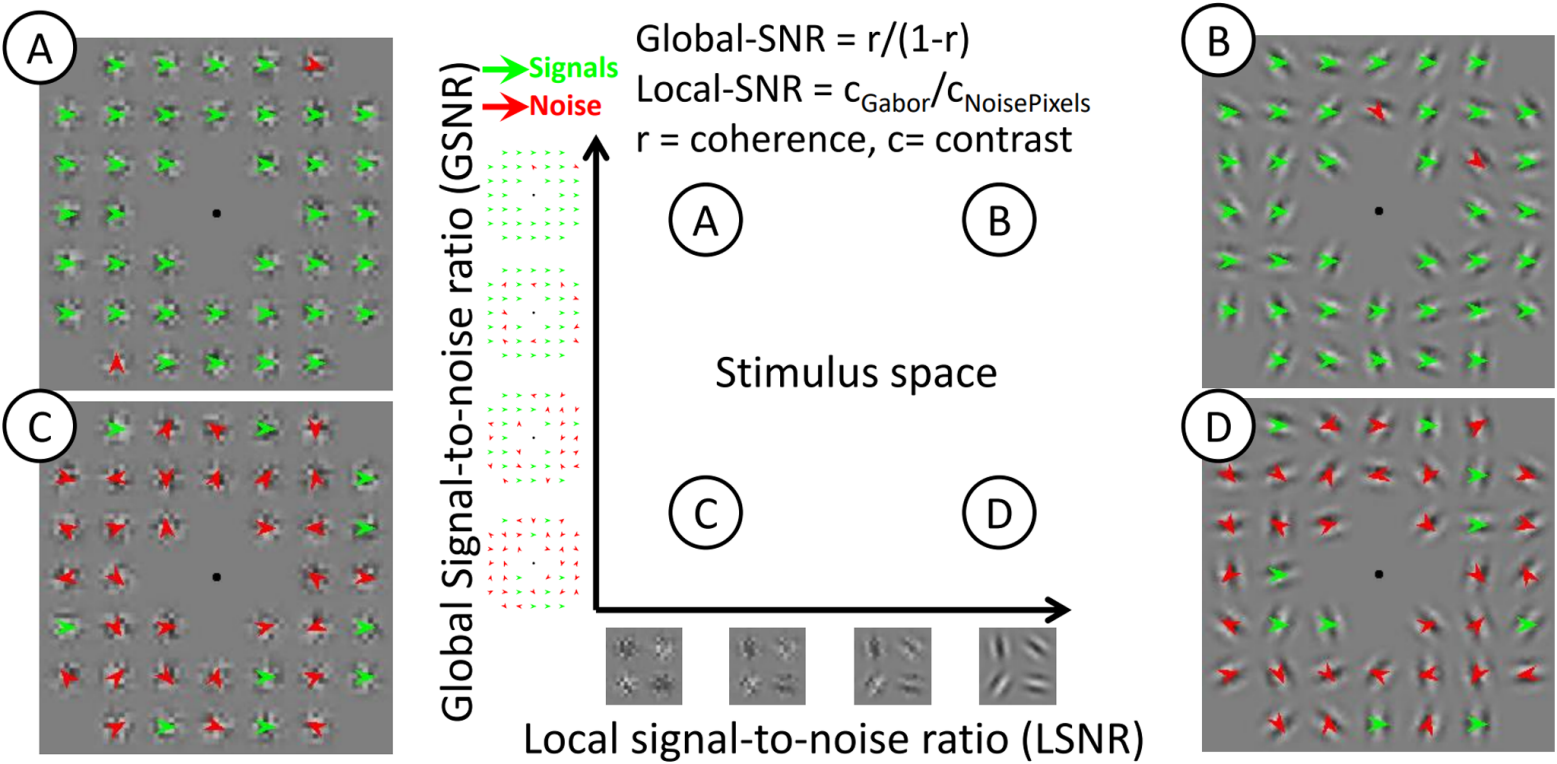
The LSNR-GSNR stimulus space. We independently manipulated the local contrast and global motion coherence to create stimuli that differed in the LSNR-GSNR space (central panel). Low LSNR corresponds to noisy elements (**A** and **C**), whereas high LSNR corresponds to clear grating elements (**B** and **D**). Low GSNR corresponds to a globally coherent motion pattern (**A** and **B**), whereas high GSNR corresponds to an incoherent motion pattern (**C** and **D**). Note that the global motion percept was rotational in Experiment 1, which is different from the rightward translational motion percept illustrated by the signal arrows (green) in this figure. Demo movies for example stimuli (including those for Experiment 2) can be found here: https://osf.io/mjtdh/.

The local-versus-global influence on confidence can be addressed by independently manipulating local and global stimulus features. Consider a stimulus containing multiple elements of Gabor gratings drifting in a common direction (the multiple-aperture array [MAA] motion pattern: see Figure 1; e.g., Amano, Edwards, Badcock, & Nishida, 2009). The observer's task was to extract the properties of the global pattern (e.g., the global motion direction). Such a multiple-element stimulus allows local and global signals to be manipulated independently. Local signal strength can be manipulated by varying the luminance contrast of individual elements. Global signal strength can be manipulated by varying the coherence of the local signals represented by the elements (which should be inversely related to between-elements variability).

To better understand how signals from local and global levels of processing influence metacognition, the present study was designed to investigate whether visual confidence changes as a function of both local and global motion signals, and how these changes influence performance by independently manipulating local and global signals in the MAA motion pattern. The strengths of local and global signals can be manipulated along a continuum in the local-global stimulus space with performance maintained at a constant level. Specifically, performance can be matched between a stimulus that has strong global, but weak local, signals (i.e., a globally-coherent motion pattern represented by noisy local elements) and a stimulus that has weak global, but strong local, signals (i.e., a globally-incoherent motion pattern represented by clear local elements). This setup allowed us to understand the separate effects of local and global signals on visual confidence judgments.

## Experiment 1

### Method

#### Participants

Initially, 35 participants (23 female and 12 male) participated in Experiment 1. They were between 18 and 24 years of age (*M* = 19.29, *SD* = 1.52), naive to the purpose of experiment, and had normal or corrected-to-normal vision. Each of them received either monetary reward or course credits as compensation for their participation. The procedure of the experiment was approved by the Sub-Committee on Research Ethics of the Research Committee of Lingnan University before data collection began, in line with the Declaration of Helsinki 2008.

To ensure that participants performed our metacognitive tasks as instructed (see *Procedure* below), we introduced two types of catch trials (very easy and very difficult ones) between which metacognitive responses were expected to differ. We excluded participants whose confidence ratings on the easy catch trials were not significantly higher than on those extremely-difficult ones. Specifically, we compared the proportion of high-confidence responses between the 30 extremely-easy catch trials and the 30 difficult catch trials. We conducted a Z test between the two proportions and excluded participants whose high-confidence responses did not significantly differ between the easy and the difficult catch trials (alpha set at .05 for each participant). We excluded 13 out of 35 participants as a result. After all, these excluded participants did not follow the instructions in giving their metacognitive responses. Importantly, the overall pattern of results remained the same even if we had included all 35 participants in the analysis.

We also tried using a less stringent criterion for excluding participants, but the general pattern of the results would remain largely the same. For details, see Section A of the Supplementary Materials. Therefore our analyses for Experiment 1 were based on 22 participants only.

#### Apparatus

The experiment was conducted with a BenQ XL2411Z monitor at a spatial resolution of 1920 × 1080 pixels (with each pixel subtending a visual angle of 0.0278°) and a refresh rate of 120 Hz. Stimuli were programmed via MATLAB Psychtoolbox 3 (Brainard, 1997; Pelli, 1997; Kleiner et al., 2007). Viewing distance was kept constant at 57 cm using a chinrest in a dimly lit room. The monitor was calibrated based on the psychophysical procedure proposed in To, Woods, Goldstein, & Peli (2013), so that the monitor's physical luminance values would be linearized according to the 256 programmable levels in the experiment program.

#### Stimuli

The MAA motion pattern adapted from Amano and colleagues (2009) was used in the present study. MAAs allow for local and global motion signals to be independently manipulated as one-dimensional (1D) and two-dimensional (2D) motion, respectively. The 1D motion refers to the local drifting motion of an individual Gabor element along the direction that is perpendicular to the orientation of the element, whilst 2D motion refers to the global motion that the drifting motion of the element represents. Figure 1 illustrates the stimulus and the independent manipulation of local and global motion signals.

The stimuli were presented as 172 randomly-oriented, drifting-Gabor elements. Each Gabor element was fitted inside a square cell within a 17-by-17 array, with each side of the square cell subtending a visual angle of 1.25° (i.e., each side of the whole 17-by-17 array subtended a visual angle of 21.25°). The spatial frequency of each Gabor element was two cycles per degree. The sigma parameter of the radially-symmetric 2D Gaussian window of the Gabor element was set to 0.25° (i.e., around 20% of the width of the square), so that the overall circular shape of the Gabor would be maintained with more than one cycle of the Gabor visible at any time.

The pattern was arranged in a 17-by-17 array of drifting Gabor elements fixed in position (centers of adjacent elements were 1.25° of visual angle apart horizontally and vertically), giving the percept of a global rotational motion. The 172 elements were presented within an invisible annulus with inner and outer radii measuring 2.5° and 8°, respectively. Total Michelson contrast for the pattern was set at a constant of 0.4.

The rotational motion pattern for Experiment 1 was created based on Lee and Lu (2010). Global motion direction was either clockwise or counterclockwise. First, each element location was assigned a motion vector (the 2D motion vector *v*) depending on whether the element was a signal or a noise element (see the *Global coherence* description below for details). Speed of each 2D motion vector (i.e., |*v*|) was kept constant at 2°/s. Then, the drifting speed for each Gabor element (the 1D motion vector, with direction always being perpendicular to the Gabor orientation and a speed of *u*) was computed as the sine component of the 2D motion vector (with direction α) relative to the orientation of the element (θ), so that the 1D drifting speed would be consistent with the 2D motion vector as if the 2D motion was represented by drifting gratings at a specific orientation viewed through an aperture (i.e., *u*  = |*v*|*sin*(α − θ)). The key manipulation of the present study was to independently vary local signals and global signals as, respectively, the contrast of the Gabor elements and motion coherence among Gabor elements (see Figure 1 for an illustration).

#### Local luminance contrast

Local signal strength was manipulated by changing the local contrast of Gabor elements. To achieve this, we first overlaid dynamic noise over each grating element, and then modulate the luminance of the element by a 2D Gaussian window to create a noisy Gabor element. As a result, the luminance value of every pixel was the sum of a signal component and a noise component (computed separately) multiplied by a factor based on the 2D-Gaussian window.

In every frame (duration: 8.33 ms), the luminance value of every contiguous noise square (4 pixels × 4 pixels, or 0.11° by 0.11° of visual angle) in a grating element was independently sampled from a uniform distribution. The range of the uniform distribution controlled the contrast of the noisy pixels and was set based on the contrast value for the noise pixels *c_N_* described below. Local noise level was determined by the local signal-to-noise ratio (LSNR), which is defined as follows:

(1) $$\begin{array}{c} LSNR\;=c_{S}\;/\;c_{N},\; \end{array}$$

where *c_S_* is the contrast value for the Gabor elements, and *c_N_* is the contrast value for the noise pixels. The Michelson's contrast value *c_S_* determined the maximum and minimum luminance values of the Gabor gratings as follows:

(2) $$\begin{array}{c} {c_{S}}_{}=\left(L_{S,\;max}-L_{S,\;min}\right)/255,\; \end{array}$$

where *L*_*S*, *max*_ and *L*_*S*, *min*_ were bounded by (*L*_*S*, *max*_ + *L*_*S*, *min*_)  = 255, so that their midpoint was equal to the luminance of the gray background. The above definition and constraint was applied in the same way to the contrast value *c_N_*, which controlled the luminance values of the noise pixels.

Signal and noise contrast values were bounded so that they summed to a constant (i.e., *c_S_* + *c_N_* = *c*), ensuring that the overall contrast of the stimulus was kept constant at *c* = 0.4 throughout the experiment. In other words, we first calculated the luminance value for each pixel of the grating element, and then jittered the luminance of each pixel based on the amount of noise set for that particular trial while maintaining the overall contrast at a constant level, and finally applied a Gaussian filter to create a noisy Gabor element.

#### Global motion coherence

Global signal strength was manipulated via varying motion coherence and was operationalized as a global signal-to-noise ratio (GSNR):

(3) $$\begin{array}{c} GSNR=n_{S}/n_{N},\; \end{array}$$

where *n_S_* and *n_N_* are, respectively, the numbers of signal and noise elements. The global 2D motion vector assigned to every signal element was computed so that it would be consistent with the rotational motion direction for that trial (either clockwise or counterclockwise). Specifically, the 2D motion direction for a signal element was tangent to a circle whose radius was the distance between the element and the center of the pattern. For every noise element, the direction of the global 2D motion vector was independently sampled from a uniform distribution within the range of (0°, 360°).

#### Overall procedure

Through the entire experiment, participants completed a perceptual and a metacognitive task at the same time on each trial. The perceptual task was a direction-discrimination task, where participants indicated whether the global rotational direction was clockwise or counterclockwise. The metacognitive task was to indicate how confident they were (high or low) in their responses in the perceptual task by wagering either 1 point (low confidence) or 10 points (high confidence). If the participant was correct in the perceptual task, the wagered point(s) would be added to their total points. If the participant was incorrect, the wagered point(s) would be deducted from the total points.

In each trial, the multiple-aperture motion pattern was presented for 250 ms (i.e., 30 frames at 120 Hz) with a fixation dot presented at the center. Participants were instructed to rest their chin on the chinrest and maintain their gaze at the fixation as steady as possible during the stimulus presentation. Then, the motion pattern disappeared and participants completed the perceptual and metacognitive tasks simultaneously by pressing one of the four keys (clockwise/counterclockwise x high/low confidence). After the keypress, feedback text reflecting their accuracy and point change (“−1” or “−10” in red for incorrect responses; “1” or “10” in green for correct responses) would be displayed centrally for 500 ms. The stimulus of the next trial would be displayed immediately afterward.

Participants completed 22 blocks of trials with 50 trials each. Each participant first went through a calibration session (eight blocks of 50 trials; 400 trials in total), followed by the main experimental session (14 blocks of 50 trials; 700 trials in total). At the end of each block, they were given additional feedback about their overall accuracy, total points accrued, and the average number of points earned per response (averaged across all responses from the beginning of the experiment until the end of that block). Participants took a break of at least 60 seconds in between every two blocks. The 22 blocks were divided into a calibration session (eight blocks; not to be included in the final analysis), followed by an experimental session (14 blocks).

#### Calibration session

**Figure 2.**
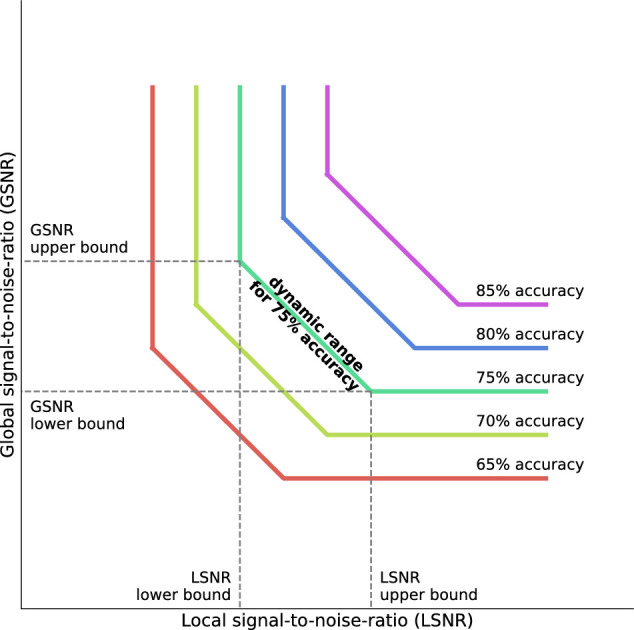
Schematic illustration of the dynamic range of local/global signals in the two-dimensional stimulus space. Colored lines represent the iso-performance lines for different levels of perceptual performance. Here, 75% (green line) is chosen to illustrate the dynamic range. The dotted lines mark the lower and upper bounds of the LSNR and the GSNR. These bounds define the dynamic range in this 2D stimulus space, within which local and global signals compensate for each other to maintain constant performance. The calibration sessions aimed at identifying the dynamic range for 75% accuracy in this 2D space by estimating the lower and upper bounds for LSNR and GSNR. Note that the iso-performance lines could be nonlinear in the actual data (depicted as linear here only for illustrations).

In the calibration session, we attempted to identify individual participants’ *dynamic range*s of stimuli in the LSNR-GSNR space for which *both* LSNR and GSNR had to vary in opposite directions (e.g., decrease in LSNR had to be compensated by an increase in GSNR) to maintain constant perceptual accuracy (75% of accuracy in the perceptual task for Experiment 1). LSNR and GSNR values were varied across trials during calibration via the accelerated stochastic approximation (ASA) algorithm (Kesten, 1958), targeting at a constant level of 75% accuracy in the perceptual task. Figure 2 illustrates the dynamic range in the 2D stimulus space. In the paragraphs below, we describe how the LSNR-GSNR dynamic range works.

When LSNR was set at the 75% contrast threshold for a participant (i.e., a noisy element: all Gabor elements almost being completely masked by noise), perceptual accuracy would be at 75% even when GSNR was set to the maximum (i.e., 100% coherent). This point, which we take as the lower bound in the LSNR dimension of the dynamic range, could be understood as the 75% contrast threshold for direction discrimination on a 100% coherently-moving global motion pattern. If LSNR decreased beyond this point, perceptual accuracy would decrease from 75% to 50% even though GSNR was kept at maximum. In other words, below the lower bound of LSNR (i.e., in Figure 2, the region to the left of the “LSNR lower bound” and above the “GSNR upper bound”), performance would be solely dependent on LSNR but independent of GSNR.

As LSNR increased from the lower bound with GSNR being held at maximum, accuracy would increase (i.e., in Figure 2, the region to the right of the “LSNR lower bound” and along the level of “GSNR upper bound“). This is because the direction-discrimination task would be easier for stimulus with higher luminance contrast. To maintain a constant accuracy level of 75%, we could decrease GSNR in compensation for the increased LSNR. We define this range of (LSNR, GSNR) values in which performance was maintained at a constant level (75% in our case) whereas both LSNR and GSNR changed inversely as our *dynamic range*.

At the other end of this dynamic range is the lower bound of GSNR, which is the 75% coherence threshold for the MAA motion pattern with suprathreshold signal contrast (i.e., a clear pattern: gratings across all elements not masked by noise). At this point, further increasing LSNR while keeping GSNR constant at its lower bound (i.e., the “75% coherence threshold” for a clear pattern; in Figure 2, the region to the right of “LSNR upper bound” along the level of “GSNR lower bound”) would have no effect on perceptual accuracy. Instead, further decreasing GSNR would decrease perceptual accuracy from 75% to 50% (i.e., the region below “GSNR lower bound” and to the right of “LSNR upper bound”), making performance solely dependent on GSNR but independent of LSNR.

The upper bounds were identified as follows. At the lower bound of LSNR (contrast threshold at 75% accuracy), perceptual accuracy would remain constant for a range of high values of GSNR (i.e., very coherent motion). We define the upper bound of GSNR as the lowest GSNR value within that high-value range. In other words, the upper bound of GSNR represents the weakest global signals that yield 75% of perceptual accuracy when local signal strength is at its threshold. The upper bound of LSNR is defined likewise.

Therefore, the purpose of the calibration session was to identify the upper and lower bounds of LSNR and GSNR, beyond which the increase in one type of signals (e.g., LSNR) could not compensate for the decrease in the other type of signal (GSNR) in maintaining constant performance.That would allow us to identify the *dynamic range* for the main experiment.

In the first half of the calibration session, we chose two blocks, either first and fourth or second and third (counterbalanced across participants) and fixed GSNR at 19 (i.e., 95% coherence), resulting in a very coherent global rotational motion). In these blocks, we let LSNR vary according to the ASA algorithm. This was to measure the LSNR threshold of the participant when global signals were the strongest. We averaged the estimates from these two staircases to obtain the LSNR threshold as the lower bound for the second half of the calibration session. In the other two blocks, we did the same but fixed LSNR at 1 (i.e., a strong local signal, resulting in very clear local Gabor elements) to obtain the GSNR threshold as the lower bound.

In the second half of the calibration session, we chose two blocks (e.g., fifth and eighth blocks) and fixed LSNR at the lower bound and let the ASA algorithm vary GSNR. This would allow us to measure the 75% GSNR threshold when LSNR was low, giving us an upper bound of GSNR. In the other two blocks, we did the same by fixed GSNR and varied LSNR to find the upper bound of LSNR. With two blocks for each of the four fixed LSNR levels, the order of the LSNR levels was randomized across blocks across participants.

#### Experimental session

In the experimental session, we created four conditions by fixing LSNR values at four specific levels, so that they were linearly-spaced between log(lower bound of LSNR) and log(upper bound of LSNR), with the lower and upper bounds estimated based on the calibration results. At each fixed LSNR level, GSNR was set to vary based on the ASA algorithm on an independent staircase. Each ASA staircase consisted of 160 trials (resulting in a total of 640 experimental trials). As these values were estimated during calibration, they varied across participants. In the following Results section, we referred to them qualitatively as “Lowest,” “Low,” “High,” and “Highest.”

In addition, we inserted 60 catch trials. In half of these catch trials (30 trials), both local and global signals were extremely strong ([LSNR, GSNR] = [1.5, 99]), making the direction-discrimination task extremely easy. Participants were expected to indicate high confidence in these easy trials. The other half of these catch trials (30 trials) were set to have extremely weak local and global signals ([LSNR, GSNR] = [0.001, 1/99]). This would make the direction-discrimination task extremely difficult and participants were expected to indicate low confidence in these easy trials. The 700 trials in the experimental session (160 trials per LSNR level × 4 LSNR levels + 60 catch trials) were randomly interleaved and then broken into 14 blocks of 50 trials, so that participants could take a break after every 50 responses.

#### Interpretation of bayes factors

In the following sections, when we interpret the values of Bayes Factors, we adopt the scale described in Jeffreys (1961), which ranks Bayes Factors in steps of half powers of 10. For a linear interpretation, we report log(BF10), which is the natural logarithms of the Bayes Factors: Negative values support the null (0) hypothesis/model and positive values support the alternative (1). The strength of the evidence then depends on the magnitude of log(BF10), as reproduced from Jeffreys (1961):

0 < | log(BF10) | < 1.15: Weak or barely-worth-a-mention
1.15 < | log(BF10) | < 2.30: Substantial
2.30 < | log(BF10) | < 3.45: Strong
3.45 < | log(BF10) | < 4.60: Very strong
4.60 < | log(BF10) |: Decisive

### Results

Prior to describing the effects of metacognition, we first verified that the stimuli generated in the experiment were indeed different across local and global signal-to-noise ratios, and that performance was matched such that we could focus on the non-performance-related effects, which reflected the major purpose of the study.

#### Stimuli differed in the LSNR-GSNR space

**Figure 3.**
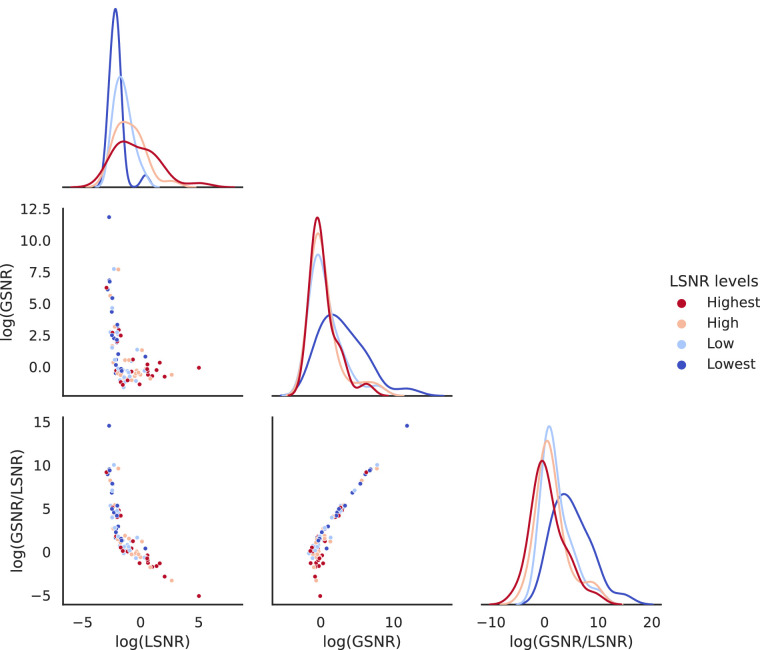
Stimuli identified by the adaptive staircase procedure for each participant in Experiment 1, varying in the two-dimensional space of LSNR and GSNR. The middle-left panel shows how GSNR (averaged over trials) varied as LSNR varied, across participants. On average, when the local elements were noisy (i.e., low in LSNR; blue and light blue dots), the global motion pattern became coherent (i.e., high in GSNR) to maintain perceptual performance. Vice versa when local elements were clear (i.e., high in LSNR; red and light red dots). To summarize this relationship, we computed log(GSNR/LSNR) as a one-dimensional variable to describe the variation in this stimulus space. The bottom-left and the bottom-middle panels show how this one-dimensional variable changes with LSNR and GSNR. respectively. The diagonal panels (from top-left to bottom-right) show the distributions of LSNR, GSNR, and log(GSNR/LSNR), respectively.

We first verified that our procedure created stimuli that were different in the LSNR-GSNR space. Figure 3 shows that stimuli differed in their local/global signal structure across the four fixed levels of LSNR. When perceptual performance was kept at a constant level, LSNR and GSNR were inversely related significantly (Pearson's rank correlation of log(LSNR) vs. log(GSNR) for the four levels of LSNR: Lowest: *r* = −0.541, *p* = 0.01; Low: *r* = −0.510, *p* = 0.02; High: *r* = −0.454, *p* = 0.03; Highest: *r* = −0.441, *p* = 0.04). The variation of the stimulus signal structure along this iso-performance curve in the LSNR-GSNR stimulus space was expressed by a single metric, log(GSNR/LSNR). Coherent but noisy stimuli (blue dots in Figure 3) would have a high log(GSNR/LSNR) value, while incoherent but clear stimuli (red dots in Figure 3) would have a low log(GSNR/LSNR) value.

A repeated-measures analysis of variance (ANOVA) revealed that there was a significant difference in log(GSNR/LSNR) across the four stimulus conditions (*F*(1.2797, 26.8731) = 17.9882, *p* < 0.001, partial η^2^ = 0.4614; with Greenhouse-Geisser correction for non-spheric within-subjects variances). In the rest of the analyses, log(GSNR/LSNR) would be used as a measurement of the separation between stimuli in the LSNR-GSNR space.

#### Matched perceptual performances across conditions

Next, we verified whether perceptual performance was matched across the four conditions by comparing their dʹ values in the direction-discrimination task. Using the signal-detection theory framework, clockwise rotation was considered as “signal-present” and counterclockwise as “signal-absent” here. Correct responses in these two types of trials were thus regarded as hit and false alarms respectively. d’ was calculated by:

(4) $$\begin{array}{ccc} & & p\left(\mathrm{Hit}\right)=p\left(\mathrm{response}=\mathrm{CW}|\mathrm{stimulus}=\mathrm{CW}\right)\\ & & p\left(\mathrm{FA}\right)=p\left(\mathrm{response}=\mathrm{CW}|\mathrm{stimulus}=\mathrm{CCW}\right)\\ & & d\text{'}=z\left(p\left(\mathrm{Hit}\right)\right)-z\left(p\left(\mathrm{FA}\right)\right)\; \end{array}$$

We implemented the following correction to avoid positive and negative infinities resulting from the z(p) function. When *p*(Hit) = 0 or *p*(FA) = 0, half a trial (0.5) would be added to the Hit or FA counts. When *p*(Hit) = 1 or *p*(FA) = 1, half a trial (0.5) would be subtracted from the Hit or FA counts (Macmillan & Creelman, 2005).

**Figure 4.**
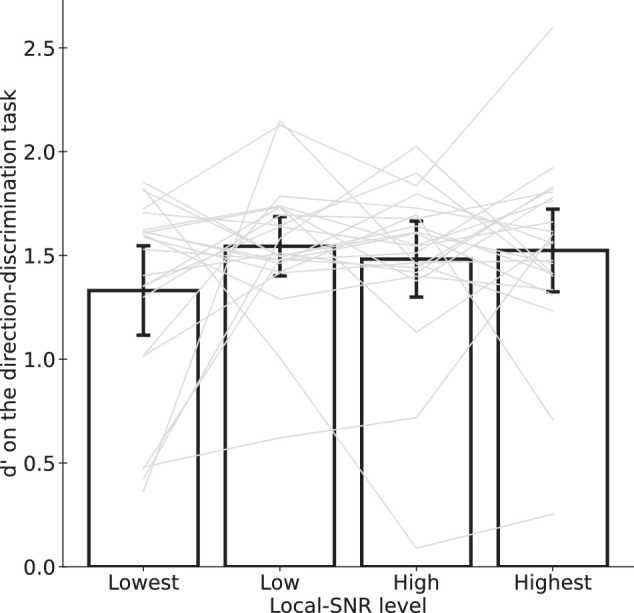
Similar perceptual performance (dʹ) across the four conditions. Each light thin line represents the data of one participant. Each bar represents the mean of each LSNR level. Error bars represent the 95% confidence intervals.

Figure 4 shows the dʹ values across the four conditions. We conducted a repeated-measures ANOVA and found no significant differences in dʹ across the conditions (*F*(3, 36) = 1.4538, *p* = 0.2357). This pattern of results are consistent with pairwise Bayesian t tests (highest log(BF10) = −0.2085 for level 1 vs. level 2, weakly favoring the null hypothesis; smallest log(BF10) = −1.4774 for level 2 vs. level 4; substantially favoring the null hypothesis). These results confirmed matched performances across conditions.

#### Effects on confidence ratings

**Figure 5.**
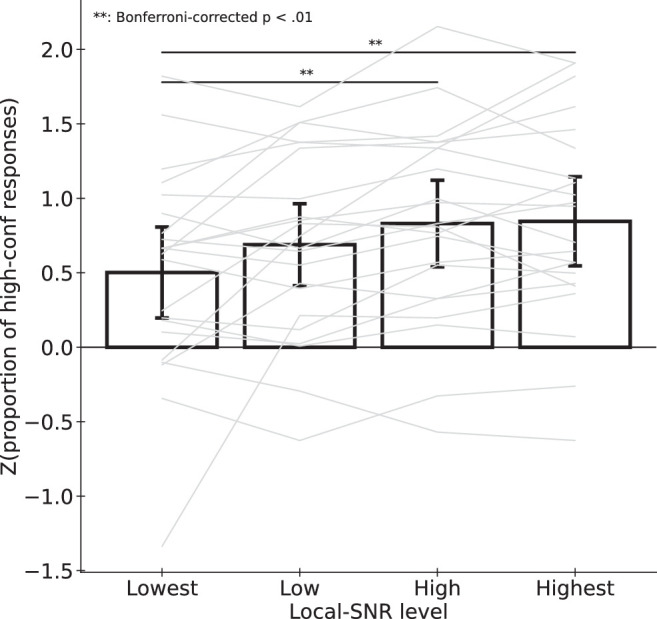
Confidence ratings increased as Local SNR increased. Each light thin line represents the data of one participant. Each bar represents the mean Z value of each LSNR level. Error bars represent the 95% confidence intervals.

To investigate the effects on metacognition, we first quantify the binary confidence responses by computing *z*(proportion of high-confidence responses) as a measurement of confidence ratings for each of the four stimulus conditions (Figure 5). Because perceptual performance was matched across the conditions, differences in confidence ratings would represent a metacognitive bias.

A repeated-measures ANOVA revealed a significant effect of stimulus condition on confidence ratings (*F*(1.6259, 34.1437) = 6.5885, *p* = 0.0061, partial η^2^ = 0.2388; with Greenhouse-Geisser correction for non-spheric within-subjects variances). Post-hoc pairwise comparisons revealed that confidence ratings for the lowest LSNR (i.e., level 1) were significantly lower than confidence rating for level 3 (*t*(21) = 3.7377, Bonferroni-corrected *p* = 0.0024; Cohen's *d* = 0.4981) and for level 4 (*t*(21) = 3.9038, Bonferroni-corrected *p* = 0.0014; Cohen's *d* = 0.5202). No significant differences were found in all other pairwise comparisons (Bonferroni-corrected *p* values ranging from 0.2277 to 1.00; Cohen's *d* from 0.0221 to 0.2825).

#### Effects on metacognitive sensitivity

**Figure 6.**
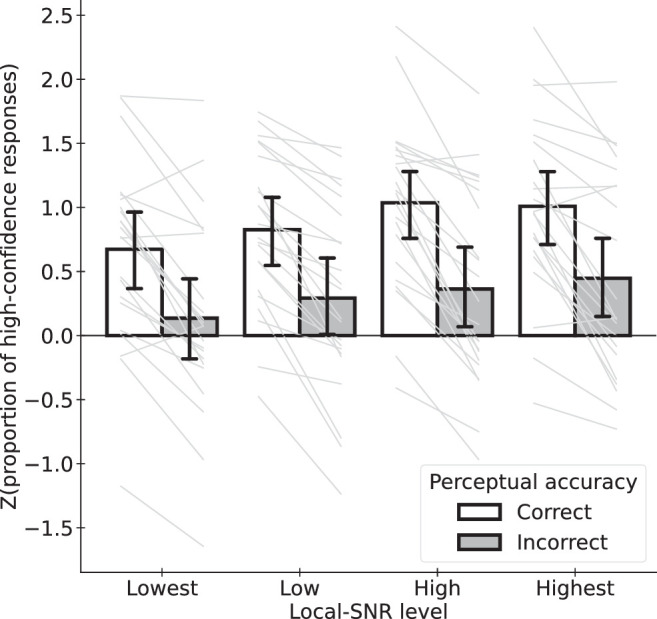
Differences in confidence ratings between correct and incorrect responses across levels of local SNR. Each light thin line represents the data of one participant. Each bar represents the mean across participants. Error bar denotes 95% confidence intervals.

Figure 6 shows the confidence ratings between correct and incorrect responses across the stimulus conditions. We measured metacognitive sensitivity as the difference in *z*(proportion of high-confidence responses) between correct and incorrect responses for the perceptual task. This represents participants’ ability to discriminate between their own correct and incorrect perceptual responses via their confidence responses.

A two-way repeated measures ANOVA (2 perceptual accuracy levels × 4 stimulus conditions) revealed a significant main effect of perceptual accuracy (*F*(1, 21) = 104.42, *p* < 0.001), suggesting that participants were, in general, able to discriminate correct and incorrect responses using confidence ratings. There was also a significant main effect of the stimulus conditions (*F*(1.8937, 39.768) = 5.612, *p* = 0.008; after Greenhouse-Geisser correction for non-spheric within-subjects variances), showing the same trend reported in the previous section about metacognitive bias. We did not find any significant interaction between perceptual accuracy and stimulus conditions (*F*(3, 63) = 0.7683, *p* = 0.5161).

We also conducted a Bayesian repeated-measures ANOVA and found that the model with only stimulus condition and perceptual accuracy as factors (i.e., main effects only without the interaction term; *p*(model | data) = 0.8422) was a better model than the model with the interaction term added (p(model | data) = 0.1219; log(BF10) = −1.9328, substantially favoring the model without the interaction term). This suggests that metacognitive sensitivity did not vary across stimulus conditions.

#### Model comparisons

How does confidence depend on local/global signal strengths in the stimulus and perceptual performance? In particular, if we partial out the effects of performance on confidence, can we observe any effects of the stimulus? To address these questions, we conducted the following model comparisons.

**Figure 7.**
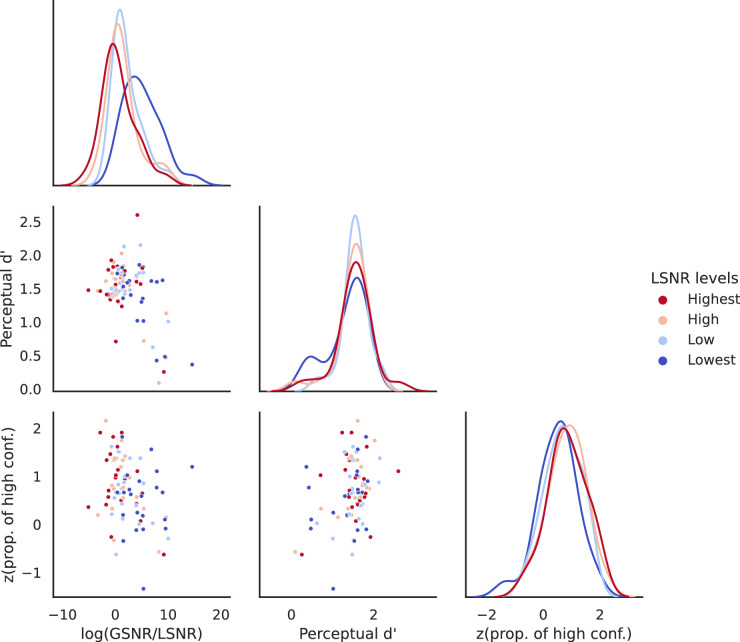
The relationship between stimulus signal structure (as log(GSNR/LSNR)), perceptual performance (as dʹ), and confidence (as z(p(high-conf))).

Figure 7 shows the distribution and the covariability of the three key variables of Experiment 1, namely, stimulus (measured as log(GSNR/LSNR)), perceptual performance (measured as dʹ), and confidence (measured as *z*(prop. High-confidence responses)). We fitted three linear mixed models to the 88 observations (22 participants × 4 stimulus conditions). All models assume that perceptual performance predicts confidence. The formulation of the three models (in model formulas using notation in *R*) are as follows:

(5) $$\begin{array}{ccc} & & \;\mathrm{Model}\;1\left(\mathrm{performance}-\mathrm{only}\right):\\ & & \;\;\mathrm{confidence}∼\mathrm{performance}+\left(1|\mathrm{participant}\right)\\ & & \;\mathrm{Model}\;2\left(\text{performance}\;\text{and}\;\text{stimulus}\right):\\ & & \;\;\mathrm{confidence}∼\mathrm{performance}+\mathrm{stimulus}\\ & & \;\;+\left(1|\mathrm{participant}\right)\\ & & \;\mathrm{Model}\;3\left(\text{performance,}\;\text{stimulus,}\;\text{and}\;\text{their}\;\text{interaction}\right):\\ & & \;\;\mathrm{confidence}∼\mathrm{performance}+\mathrm{stimulus}\\ & & \;\;+\mathrm{performance}:\mathrm{stimulus}+\left(1|\mathrm{participant}\right)\; \end{array}$$

The “(1 | participant)” term denotes a random intercept for each participant, so that individual differences in confidence judgments would be accounted for in all models.

We computed the Bayesian information criterion (BIC) for each of these three models:

BIC for Model 1 = 101.646
BIC for Model 2 = 89.592
BIC for Model 3 = 88.799 (lowest among all three models)

The result shows that the model with the stimulus terms (Models 2 and 3) are better than the model without the stimulus term (Model 1) in explaining the variability of confidence judgments. Using the approximation of Bayes Factors based on BIC: log(BF10) = 0.5*(BIC of Model 0 − BIC of Model 1)), we evaluated the evidence for model comparisons as follows. Model 2 versus Model 1: log(BF21) = 6.027, indicating decisive evidence favoring Model 2 over Model 1; Model 3 versus Model 1: log(BF31) = 6.4235, indicating decisive evidence favoring Model 3 over Model 1. We found very weak evidence favoring Model 3 over Model 2 (log(BF32) = 0.3965). Because both Models 2 and 3 contain stimulus as a predictor, our results support that stimulus is an important factor in influencing confidence even after accounting for the effect of performance on confidence.

We performed two additional analyses to evaluate the relationship among stimulus, performance, and confidence. First, we found a positive correlation (*r* = 0.58, *p* = 0.0047) between the stimulus effects on performance and the stimulus effects on confidence across participants. This offers an explanation for our findings in Experiment 1 from the individual-difference perspective because it suggests that participants whose performance was more strongly affected by stimulus were also strongly influenced by stimulus on their confidence.

Second, we conducted a within-subjects mediation analysis showing that stimulus directly influenced confidence and performance did not provide significant mediation. For further details of these analyses, please refer to Section B of the Supplementary Materials.

#### Discussion

Results in Experiment 1 show that, even at matched perceptual performance, varying the local/global signal structure of stimulus could result in difference in metacognitive responses. Specifically, confidence ratings were found to be lower when local signals were weak and global signals were strong (noisy but coherent) than when local signals were strong and global signals were weak (clear but incoherent).

However, this pattern of results could be due to the binary, high-low options of confidence and the yes-no type of perceptual task (e.g., Lee, Ruby, Giles, & Lau, 2018). In Experiment 2, we used the same multiple-aperture motion pattern but a completely different direction-judgment task, in which participants had to reproduce the perceived direction by providing responses on a continuous scale instead of making a binary choice. This would increase the granularity of both the perceptual and confidence responses and would allow us to test whether the pattern of results in Experiment 1 would still hold for a different task setting.

## Experiment 2

In Experiment 2, we aimed at testing the same effects we found in Experiment 1 using an analog task. Motion direction became translational (instead of rotational). The perceptual task was to reproduce the perceived global motion direction by turning a simulated dial on the screen. Participants then indicated their uncertainty by making a wager on an “error range”: they traded wagering points for the width of the range, so that a wider range would lead to a fewer wagering points and vice versa. The width of this “bet span” would be taken as an inverse measure of confidence (wider means less confidence).

### Method

#### Participants

Participant recruitment follows the same protocol as in Experiment 1, and 35 participants (20 females; age range: 19–24 years, *M* = 19.14, *SD* = 1.38; none participated in Experiment 1) performed Experiment 2. Using the same procedure as in Experiment 1, the confidence responses of six participants did not show significant differences between the easy and difficult catch trials (*p* values from 0.10 to 0.92). Therefore we only included these 29 participants (*p*s < 0.02) in the analyses of Experiment 2 without changing the overall pattern of results. Similar to Experiment 1, we tried using another exclusion criterion, but the results for Experiment 2 remained the same. See Supplementary Materials for details.

#### Apparatus

The apparatus was identical to Experiment 1.

#### Stimuli

**Figure 8.**
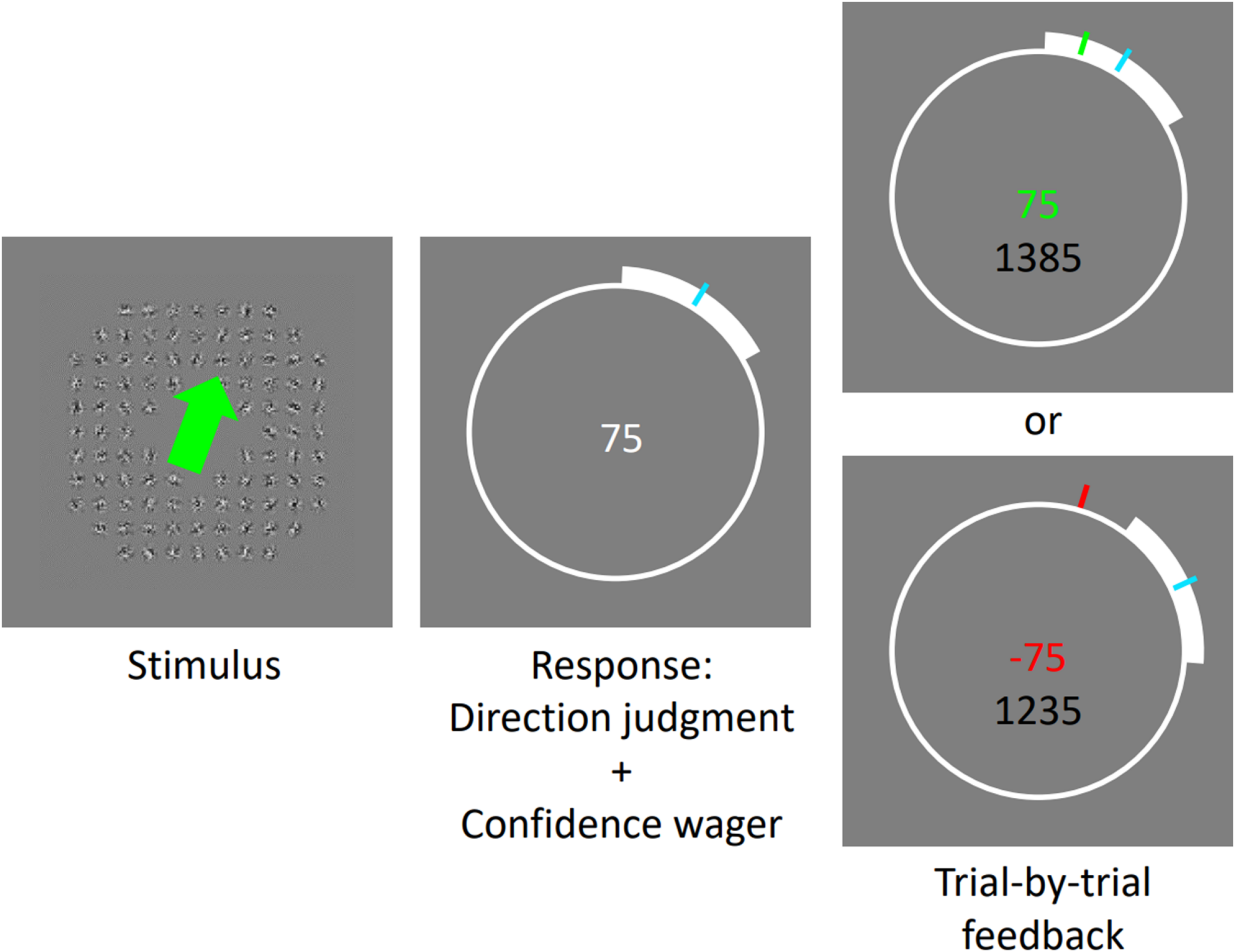
Procedure of Experiment 2. Participants first saw the multiple-aperture motion pattern with a translational global motion direction. Then they indicated the perceived global motion direction by turning a simulated dial (cyan pointer) using the computer mouse. Before confirming this direction-judgment response, participants adjusted the width of the bet span (thick white bar; symmetrical around the cyan pointer) so that the bet span would cover the global motion direction of the stimulus. The white number at the center (“75”) represents the wagering points and was updated in real time as participants adjusted the width of the bet span (wider bet span, lower wagering points). After the participants had submitted their response, trial-by-trial feedback would then be given, depending on whether the response was correct (top-right panel, with white bar covering the true stimulus direction indicated in green; point increment for this trial in green; total points so far after this increment in black) or incorrect (bottom-right panel, with white bar not covering the true stimulus direction indicated in red; point deduction for this trial in red; total points so far after deduction in black). For demo movies of the same example stimuli, please visit: https://osf.io/mjtdh/.

Details of the stimulus were identical to those in Experiment 1, except for the global motion direction. Instead of rotational motion, the global motion direction was translational in Experiment 2 (see Figure 8). In every trial, the signal global motion direction was uniformly sampled from 0° to 360°.

#### Procedure

Figure 8 illustrates the procedure of Experiment 2. The perceptual task was to indicate the perceived global motion direction of the visual stimulus by adjusting an on-screen dial using the computer mouse. To indicate their confidence, participants adjusted the width of a bar that spanned around the perceived direction, so that the span could include the actual stimulus direction. To gamify the experiment, bar width was traded for wager points (i.e., a longer bar wagered fewer points and vice versa [cf., Yoo, Klyszejko, Curtis, & Ma (2018]). This “bet span” was taken as a measurement of confidence. Participants were instructed to respond with a narrow range if they were confident in their answer. Conversely, participants could respond with a wider bar width if they were less confident. In every trial, if the bet span covered the actual stimulus direction, they received the points they wagered. If they were incorrect, points would then be deducted according to how much they bet. To keep participants engaged, trial-by-trial feedback was provided throughout the experiment, and participants were shown their average earned points per trial.

There were no calibration sessions in Experiment 2. LSNR was fixed at three levels: 0.15, 0.30, 0.60, which were determined based on a few pilot participants (not included in the data reported here). As in Experiment 1, GNSR was varied independently via the ASA algorithm (Kesten, 1958) at 75% accuracy for each fixed LSNR level. Because there was no absolute definition of correctness in the direction-judgment task, we defined correctness based on the magnitude of the response error: a correct response on every trial was defined as the response direction falling within 45° of the stimulus direction (i.e., correct if absolute error was no more than 45°; incorrect otherwise).

Each participant completed a total of 500 trials, which were split into 20 blocks of 25 trials. There were 150 trials for each fixed LSNR level. To ensure that participants performed the tasks as instructed, there were 25 easy catch trials with [LSNR, GSNR] = [1.5, 999], and 25 difficult catch trials with [LSNR, GSNR] = [0.001, 1/999]. All trial types (LNSR levels and the catch trials) were randomly interleaved and then grouped into 20 blocks of 25 trials. In addition to trial-by-trial feedback, a final feedback showing the total points accumulated by the participant and their average points earned per trial was provided at the end of each block.

### Results

To quantify the task performance, we estimated the perceptual precision in global motion direction judgment based on the following analysis. First, we computed the direction-judgment error by subtracting the stimulus direction from the response direction for every trial, resulting in values ranging from −360° to 360°. We then wrapped resulting values around so that every direction-judgment error value would fall within the range of (−180°, 180°].

**Figure 9.**
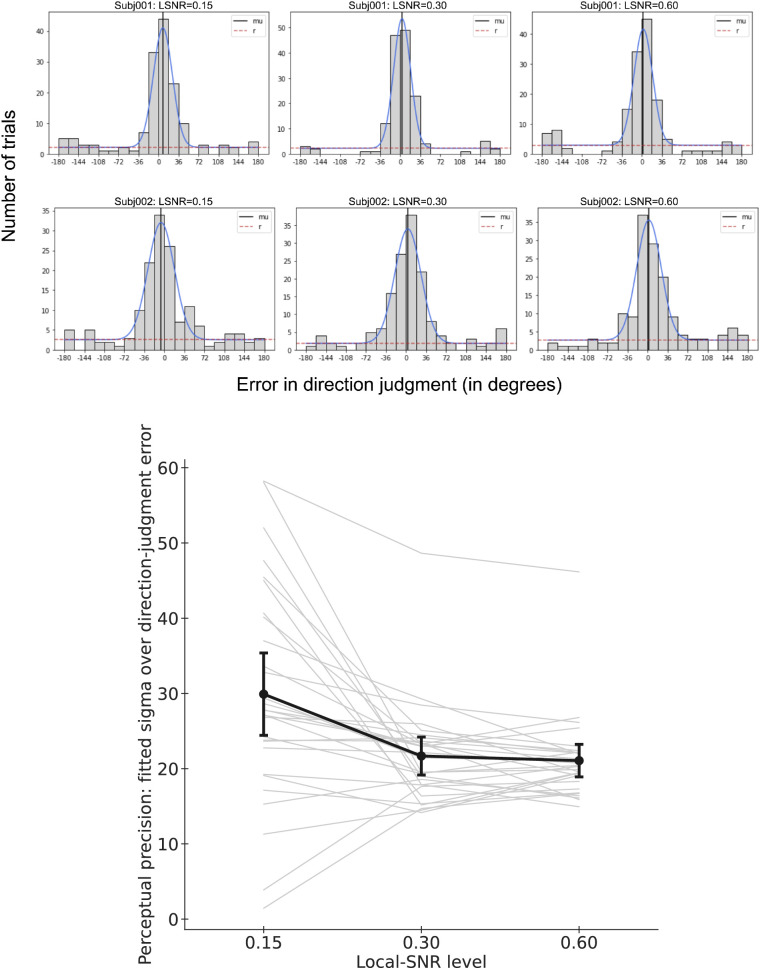
Perceptual performance for each stimulus condition measured as precision in the direction-judgment task. (a): Illustration of the uniform-normal mixture fit (blue curve) to the distribution of the direction-judgment errors (20 bins of 36° each) for two participants in the three local-SNR level conditions. (b) Perceptual performance (measured inversely using perceptual precision) was worst when LSNR was the lowest (level 1). Each light thin line represents the data of one participant. The dark thick line represents the mean. Error bars represent the 95% confidence intervals.

Finally, for each participant, we fitted a Uniform-Normal mixture (UNM) model to the direction-judgment error values. The probability density function of the UNM is defined as:

(6) $$\begin{array}{ccc} & & y_{UNM}\left(x;\;\omega ,\;\mu ,\;\sigma^{2},\;a,\;b\right)\;\\ & & =\;\omega N\left(x;\;\mu ,\;\sigma^{2}\right)+\left(1\;-\;\omega \right)U\left(x;\;a,\;b\right)\; \end{array}$$

where *N* is the normal distribution over *x* with μ and σ being the mean and sigma of the normal distribution, respectively, and *U* is the uniform distribution with the lower and upper bounds *a* and *b* being set to −180 and 180, respectively. ω, being limited to be within [0, 1], is the weight assigned to the normal distribution in the mixture of the UNM model. Direction-judgment error resulting from blind guessing should result in a fitted ω value being close to 0. We took the fitted σ value for each participant in each condition as an inverse measurement of perceptual precision (i.e., smaller σ means higher precision). The top panel in Figure 9 shows an example fit of two participants’ data across the three stimulus conditions.

#### Perception performance measured as precision in direction judgment

The bottom panel of Figure 9 shows the perceptual performance (measured as precision) varied across stimulus conditions, as revealed by a one-way repeated-measures ANOVA (*F*(2, 56) = 11.7834, *p* = 0.0001). Performance was worst when local SNR was the lowest (LSNR = 0.15; *M* = 29.9058, *SD* = 14.3923; LSNR = 0.30: *M* = 21.6825, *SD* = 6.6409; LSNR = 0.60: *M* = 21.0679, *SD* = 5.6846; Comparisons: “0.15 vs. 0.30”: t(28) = −3.4501, *p* = 0.0018; “0.15 vs. 0.60”: t(28) = −3.5145, *p* = 0.0015). There was no significant difference in perceptual precision between the two higher LSNR conditions (“0.30 vs. 0.60”: t(28) = −0.9781, *p* = 0.3364).

#### Metacognitive sensitivity

**Figure 10.**
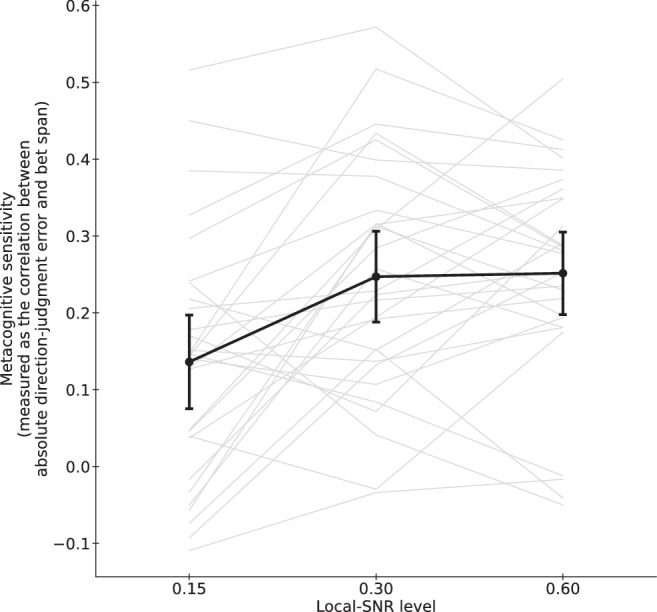
Lower mean confidence response for 0.15 LSNR, but response reached a plateau for 0.30 and 0.60 LSNRs. Each light thin line represents the data for one participant. The dark thick line represents the mean. Error bars represent the 95% confidence intervals.

To evaluate metacognition sensitivity, we computed the Fisher's *Z* transformation of the Spearman's rank correlation between the absolute value of direction-judgment error (larger = worse performance) and bet span (wider = less confident) across trials. Higher Fisher's *Z* represents higher metacognitive sensitivity. Figure 10 shows the metacognitive sensitivity across the three conditions.

Unlike in Experiment 1 (Figure 4), the metacognitive sensitivity was lower when the local SNR was low. A repeated measures ANOVA suggested significance of the perceptual precision and confidence (*F*(2, 56) = 11.0587, *p* < 0.0001). Paired *t*-tests indicated that metacognitive sensitivity was lower when LSNR = 0.15 (*M* = 0.1362, *SD* = 0.1604) than when LSNR = 0.30 (*M* = 0.2471, *SD* = 0.1555; *t*(28) = 3.8274, *p* = 0.0007) and when LSNR = 0.60 (*M* = 0.2516, *SD* = 0.1411; *t*(28) = 3.6934, *p* = 0.0009). Again, we did not find any significant difference between LSNR = 0.30 and 0.60 (*t*(28) = 0.1983, *p* = 0.8443). This pattern of results was expected because perceptual performance was lower when LSNR was low (see Figure 9).

#### Metacognition bias: Lower confidence for noisier stimulus

Because perceptual performance was not equated across the three LSNR conditions, we matched performances across the three LSNR conditions by focusing on the trial-by-trial direction-judgment error. Here, we quantified the perceptual performance of one trial by computing the absolute value of the direction-judgment error. A small absolute error value represents a high precision, which was taken as a representation of good perceptual performance (and vice versa for large absolute error values).

We then matched perceptual performances across the 3 stimulus conditions by sorting absolute error values into 8 bins with equal width of 22.5° from 0° to 180°. The first bin contained responses with absolute error within the range of [0°, 22.5°), which were closest to accurate responses (good perceptual performance). The last bin [157.5°, 180°) would represent responses that were farthest away from the presented global stimulus direction. As a result, the eight bins represent eight levels of perceptual performance.

**Figure 11.**
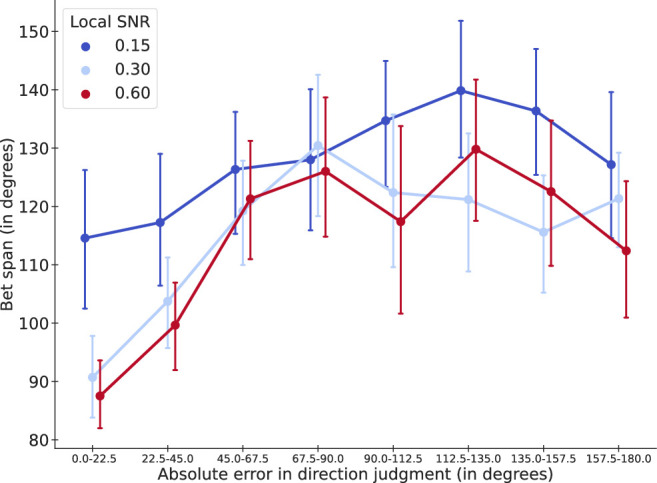
Lower SNR yields less confidence regardless of the precision of the response. Mean bet span for 0.15 SNR is significantly higher than 0.30 and 0.60 SNR; the bet span is interpreted as inversely related to confidence.

Figure 11 showed the averaged width of bet span across different levels of performance. In general, bet span was wider when LSNR = 0.15 than when LSNR = 0.30 and 0.60 (blue line above orange and green lines), and bet span increased in width as performance got worse (an overall increasing trend of all three lines).

We conducted a 3 × 8 repeated-measures ANOVA, with three LSNR levels and eight performance levels as the factors. Because the within-subjects variances were significantly non-spherical (p values for Mauchly's tests ranging from <0.0001 to 0.0014; Greenhouse-Geisser epsilon ranging from 0.46 to 0.64), the degrees of freedom for the *F* tests below were done with Greenhouse-Geisser correction.

We found a significant main effect of LSNR level (*F*(1.2821, 29.4894) = 8.9478, *p* = 0.0032), suggesting that bet span was wider (i.e., confidence was lower) when local SNR was lower. We also found a significant main effect of performance levels (*F*(4.2745, 98.3136) = 18.2147, *p* < 0.0001), suggesting that bet span generally became wider as performance got worse (i.e., confidence correlated with performance). We did not find a significant interaction between LSNR level and performance (*F*(6.4556, 148.4792) = 1.3445, *p* = 0.2375).

#### Model comparisons

**Figure 12.**
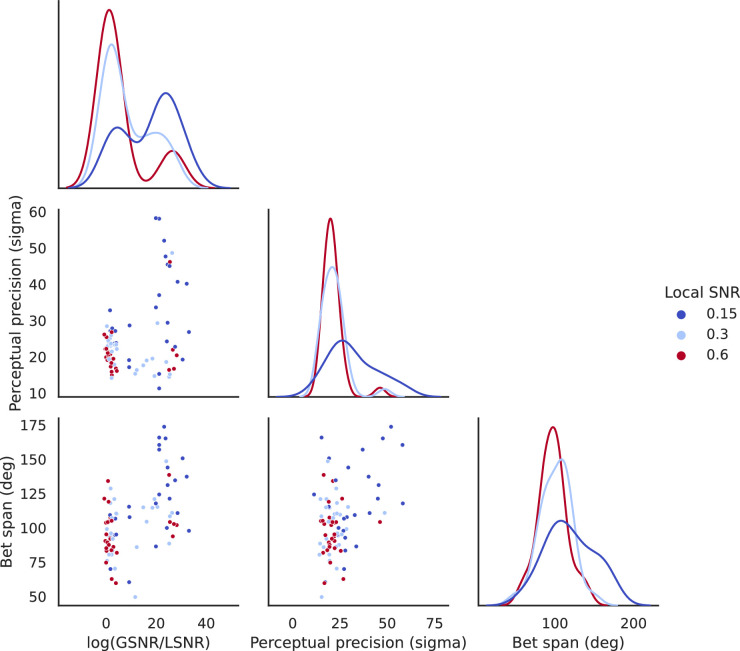
The relationship between local/global signal structure in the stimulus (as log(GSNR/LSNR) ), perceptual performance (as precision estimated by sigma), and confidence (as the width of bet span).

Figure 12 shows the same relationship among stimulus (represented in log(GSNR/LSNR)), perceptual performance (as the precision in direction judgment, measured (inversely) using sigma_hat in Figure 9), and confidence (inversely measured based on bet span; avg_betspan) as in Figure 7 for Experiment 1.

We performed similar model comparisons as in Experiment 1. We fitted three linear mixed models, with the assumption that performance predicts confidence, to the 87 observations (29 participants × 3 stimulus conditions). The three models we compared were the same as those we set up for Experiment 1, namely, Model 1 (performance-only), Model 2 (performance and stimulus), and Model 3 (performance, stimulus, and their interaction), with random intercept included for each participant for each model, so that individual differences in confidence judgments would be accounted for in all models. More formally, the three models are

Model 1 (performance-only): bet span ∼ precision + (1 | participant)
Model 2 (performance and stimulus): bet span ∼ precision + stimulus + (1 | participant)
Model 3 (performance, stimulus, and their interaction): bet span ∼ precision + stimulus + precision:stimulus + (1 | participant)

We computed the BIC for each of these three models as follows:

BIC for Model 1 = 900.02
BIC for Model 2 = 883.70 (lowest among all three models)
BIC for Model 3 = 884.98

The result is similar to that in Experiment 1: Models 2 and 3, both of which contain the stimulus term, are better than Model 1 (Model 2 vs. Model 1: log(BF21) = 8.16, indicating decisive evidence favoring Model 2 over Model 1; Model 3 vs. Model 1: log(BF31) = 7.52, indicating decisive evidence favoring Model 3 over Model 1). There is only weak evidence favoring Model 2 over Model 3 (log(BF23) = 0.64. The pattern of results is generally similar to that in Experiment 1.

We performed the same follow-up analyses as we did for Experiment 1 on the data from Experiment 2. First, unlike in Experiment 1, we did not find any significant correlation between the stimulus effects on performance and the stimulus effects on confidence across participants (*r* = −0.049, *p* = 0.80).

Second, in a mediation analysis similar to that for Experiment 1, we again found a significant direct effect of stimulus on confidence, although performance significantly mediated the influence of stimulus on confidence (i.e., a partial mediation). In summary, in Experiment 2, stimulus exerted its effects both directly on confidence and indirectly via performance. For details of these two additional analyses, please refer to Section B of the Supplementary Materials.

## General discussion

In two experiments with different task settings, we found that visual confidence in motion direction judgments depended on the local/global signal structure of the stimulus after controlling for the effects of perceptual performance.

Specifically, we found a systematic effect of the trade-off between local and global signal strength on confidence judgments: participants were more confident on a stimulus with strong local signal but weak global signals (i.e., clear but incoherent) than on a stimulus with weak local signal and strong global signal (i.e., noisy but coherent), despite equal performance between the two situations.

Results from the model-comparison analyses suggest that the local/global signal structure of the stimulus is an important factor in predicting confidence, even after the effects of perceptual performance have been taken into consideration.

### Evidence versus variability

The present study is similar to previous ones in which evidence and variability in the stimulus were manipulated independently (e.g., de Gardelle & Mamassian, 2015, Zylberberg et al., 2014; Boldt et al., 2017; Spence et al., 2016). In those studies, evidence was often defined as the distance between the mean across the elements (e.g., the averaged direction over multiple moving dots in a random-dot kinematogram) and the perceptual decision criterion (e.g., perceived upward direction).

However, in the present study, instead of varying the evidence, we directly manipulated two types of signals, with the intention of varying the activation at different levels of visual processing and observing the effects on confidence responses. To our knowledge, the present study is the first to independently manipulate stimulus uncertainty at both the local and global levels of visual processing.

In particular, Zylberberg et al. (2014) found that confidence for an orientation-averaging task increased with orientation variability across multiple line segments, even when performance in high-variability conditions was worse than that in low-variability conditions. This finding is similar to our results showing that confidence was higher when global coherence was low (high-variability) than when it was high (low-variability). However, the difference between Zylberberg et al. and the present study was that we held performance constant by balancing sensory signals at the local and global processing levels, which is similar to the equivalent-noise manipulation (e.g., Dakin, Mareschal, & Bex, 2005). In their paradigm, local-level noise and global-level noise are pitched against each other as in the present study. Nevertheless, it remains unclear how uncertainty is represented at the decision level for this type of equivalent-noise stimulus. Future studies can explore different ways to represent uncertainty at multiple stages of processing and test different perceptual-decision models.

In general, previous studies have shown that visual confidence depends on variability in the stimulus even when perceptual performance is held constant (e.g., Spence et al., 2016; Boldt et al., 2017). They found that confidence decreased when stimulus variability was increased. Based on this pattern of results, if we just consider the effect of variability of local motion directions, a globally-coherent motion pattern could have resulted in higher confidence as it should have a lower direction variability than an incoherent one. However, we found exactly the opposite in the present study. This suggests that visual confidence may be more heavily influenced by factors other than variability in a stimulus ensemble.

### Possible explanations

Our findings suggest that physical properties of a stimulus could lead to a systematic bias in confidence judgments even when perceptual performance was held constant. This is different from the findings in earlier studies in which visual confidence was found to track performance and remained relatively constant despite changes in stimulus features (e.g., Barthelme and Mamassian, 2010). Our findings are consistent with Bertana et al. (2021) in that confidence could be biased by visual cues extracted from physical properties of the stimulus (Bertana et al., 2021). Following Bertana et al.’s model, it is possible that the metacognitive system “perceives” local signals to be stronger than global signals, resulting in an overall higher confidence rating when the local signal was stronger.

One possible explanation for the observed effect on metacognitive bias lies on the difference in levels of visual processing at which the induced noise suppresses neural signals. Stimuli with stronger local signals (i.e., clearer images) should generate stronger direction-specific activation in early, local stages of motion processing than stimuli with weaker local signals (i.e., noisier images). On the contrary, stimuli with stronger global signals (i.e., more coherent) should generate stronger direction-specific activation in later, global stages of motion processing than stimuli with weaker global signals (i.e., more incoherent).

We propose that different weights may be assigned to local and global signals by a high-level metacognitive system in the evaluation of information. The confidence rating measured on a perceptual response may represent the metacognitive system's evaluation of the amount of information available for completing the perceptual task. Our findings may then imply that the metacognitive system, at a constant level of perceptual performance, evaluates low-level signals (e.g., luminance contrast) as more informative than high-level signals (e.g., global coherence). Previous studies have reported similar unequal weighting of visual information within the motion processing hierarchy during motion adaptation (Lee, 2018) or over time during confidence integration (Lee, de Gardelle, & Mamassian, 2021).

This unequal weighting between low- and high-level signals by the metacognitive system may also help further characterize the positive-evidence bias (PEB) reported in previous studies (e.g., Koizumi, Maniscalco, & Lau, 2015; Maniscalco, Peters, & Lau, 2016; Odegaard et al., 2018; Peters et al., 2017; Samaha, Barrett, Sheldon, LaRocque, & Postle, 2016; Samaha & Denison, 2022; Samaha, Switzky, & Postle, 2019). The PEB is a phenomenon in which evidence in favor of the correct option is given a heavier weight by the metacognitive system during confidence judgments. Importantly, our study has demonstrated the possibility that a single stimulus can contain both positive and negative evidence together (i.e., clear Gabor with low coherence, noisy Gabor with high coherence). If the metacognitive system evaluated luminance contrast and global coherence equally as sensory evidence, the PEB would have predicted similar confidence ratings between noisy-coherent and clear-incoherent motion patterns. Our findings support the opposite, as we found a confidence bias in favor of local luminance contrast over coherence in the present study. Such a metacognitive bias suggests that low-level signals may contribute more to sensory evidence for confidence computation than high-level signals do.

Another possible mechanistic explanation is the suboptimal placement of decision criteria for confidence judgments (Shekhar & Rahnev, 2021; Rahnev & Denison, 2018). For example, the observer could be using a rigid set of decision criteria across different amounts of noise in the stimulus, resulting in higher confidence for the stimulus with greater variability despite constant or even worse perceptual performance (Zylberberg et al., 2014). Because we found higher confidence for lower global coherence, which was represented as greater variability in local motion directions across elements in the MAA motion pattern, our findings are consistent with Zylberberg et al.’s rigid-criterion model.

Last, the task nature is unlikely to be an explanation for our results. The stimulus-dependent bias in confidence judgments was observed in both experiments, in which we used different tasks. For instance, both the perceptual decision and the confidence rating were binary in Experiment 1, whereas they were continuous in Experiment 2. It is possible that such bias in confidence could be related to stages of processing that are beyond sensory processes and perceptual decision-making.

### Limitations

In the present study, visibility could be a potential confound with local signals. Because noise was defined as “local” when Gabor elements were masked by dynamic noisy pixels, a MAA motion pattern with high local noise would essentially be low in visibility. With this interpretation, Rausch, Hellmann, & Zehetleitner (2021) weighted-evidence-and-visibility model could potentially explain the present findings because our local-noise manipulation affected visibility of the stimulus, which in turn affected confidence without affecting performance.

Another alternative interpretation of our findings is that the local noise at the early stages of motion processing would eventually be propagated to and could influence later, global stages of processing. As a result, local noise could have a greater impact on confidence than global noise, which could explain the present findings. Nonetheless, we limited the effect of such propagation of noise on perceptual performance by matching performance across conditions, and still found systematic differences of confidence responses. It is possible that this “hierarchical interpretation” of the relationship between local and global noise could be a reason for the metacognitive system to assign heavier weights to local stages of processing when evaluating the available information for a task.

The use of adaptive staircase procedure to control for performance could have affected confidence judgments in certain ways. For instance, in Experiment 2, perceptual performance was not perfectly matched across the three stimulus conditions. One of the reasons is that, given the continuous nature of the responses in the direction-judgment task, there is no objective way to define a “correct” response for the staircase procedure. Another drawback is that staircase procedures could inflate the estimates of metacognitive sensitivity (Rahnev & Fleming, 2019). However, for both experiments, we attempted to control for the effects of performance by including performance in all models for comparison. Also, our main conclusion focuses on the bias in confidence judgments, which would be less susceptible to the inflation by staircase procedures than metacognitive sensitivity would. Future studies could use fixed levels of difficulty to assess the effects of local/global signals on metacognition.

### Conclusions

We found that physical properties can affect metacognition despite matched performance. Confidence was found to be higher on the stimuli with strong-local/weak-global signals than on those with weak-local/strong-global signals. This may imply that the metacognitive system weighs signals coming from early processing stages more heavily than later stages when evaluating for information available for a visual task.

## Supplementary Material

Supplement 1
