# Supplementary material for: Metacognitive bias resulting from trade-off between local and global motion signals
Source: J Vis. 2023 Sep 11;23(10):7. doi: 10.1167/jov.23.10.7 (PMC10501489; doi:10.1167/jov.23.10.7)
Supplement: Supplement 1 [file jovi-23-10-7_s001.docx]

# SUPPLEMENTARY MATERIALS

## A. A different participant-exclusion criterion

We have attempted to use a different participant-exclusion criterion for both experiments, which would be less stringent than the existing criterion of “significantly higher confidence in easy catch trials than hard ones”. This new criterion would be to exclude participants who did not produce “positive correlation between confidence and accuracy”.

For Experiment 1, we computed Type-2 d’ across all trials for every participant because a positive Type-2 d’ captures the meaning of a “positive correlation between confidence and accuracy” for binary perceptual and confidence responses. This new criterion gave us n=33 out of the 35 participants who completed the task, compared with our original criterion that gave us n=22 out of 35.

We reran all the statistical analyses for Experiment 1, and found that the conclusions from all statistical tests for each subsection would stay the same as the n=22 sample, except that the differences in a couple of specific tests became insignificant with the n=33 sample (e.g., under subsection 1 of Results, the correlation of log(LSNR) and log(GSNR) became insignificant for Low LSNR level (p changed from .02 to .17) and High LSNR level (p changed from .03 to .23).

For Experiment 2, we implemented the new criterion by correlating the absolute angular error in the direction-judgment responses with the bet span and removed participants whose correlations were not positive. This led us to n=32 out of 35, compared with our original criterion that gave us n=29 out of 35. For Experiment 2, results of all statistical analyses are consistent between the new and our original criteria.

##

## B. Follow-up analyses on the stimulus-performance-confidence relationship

### Correlation between stimulus effect on performance and stimulus effect on confidence

For Experiment 1, we estimated the stimulus effect on perceptual performance using the simple linear regression slope of perceptual d’ on log(GSNR/LSNR) over the four levels of LSNR. Similarly, we estimated the stimulus effect on confidence using the simple linear regression slope of z(proportion of high-confidence responses) on log(GSNR/LSNR) over the four levels of LSNR. Figure S1 shows the scatter plot of the stimulus effect on confidence on the stimulus effect on perceptual performance. We found a significant positive correlation between the two effects for Experiment 1 (r = .58, p = .00473).


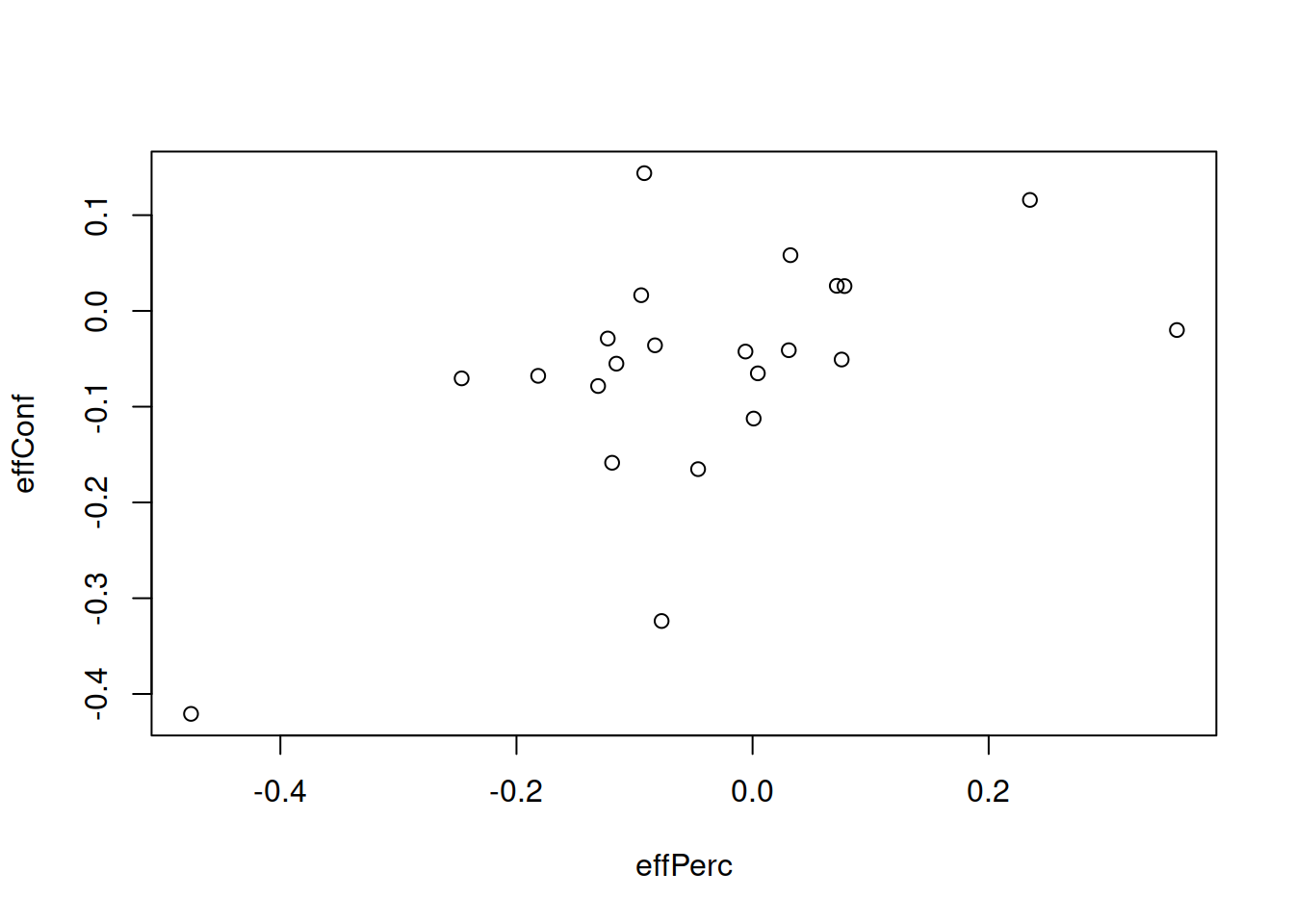


Figure S1. Scatter plot of the stimulus effect on confidence (vertical axis) over the stimulus effect on perceptual performance (horizontal axis) for Experiment 1.

For Experiment 2, we estimated the stimulus effect on perceptual performance using the simple linear regression slope of perceptual imprecision (the fitted sigma value described under Results of Experiment 2; an inverse measurement of performance) on log(GSNR/LSNR) over the three levels of LSNR. Similarly, we estimated the stimulus effect on the lack of confidence using the simple linear regression slope of the average bet span (an inverse measurement on confidence) on log(GSNR/LSNR) over the three levels of LSNR. Figure S2 shows the scatter plot of the stimulus effect on confidence on the stimulus effect on perceptual performance. The correlation between the two effects for Experiment 2 was insignificant (r = -.049, p = .80).


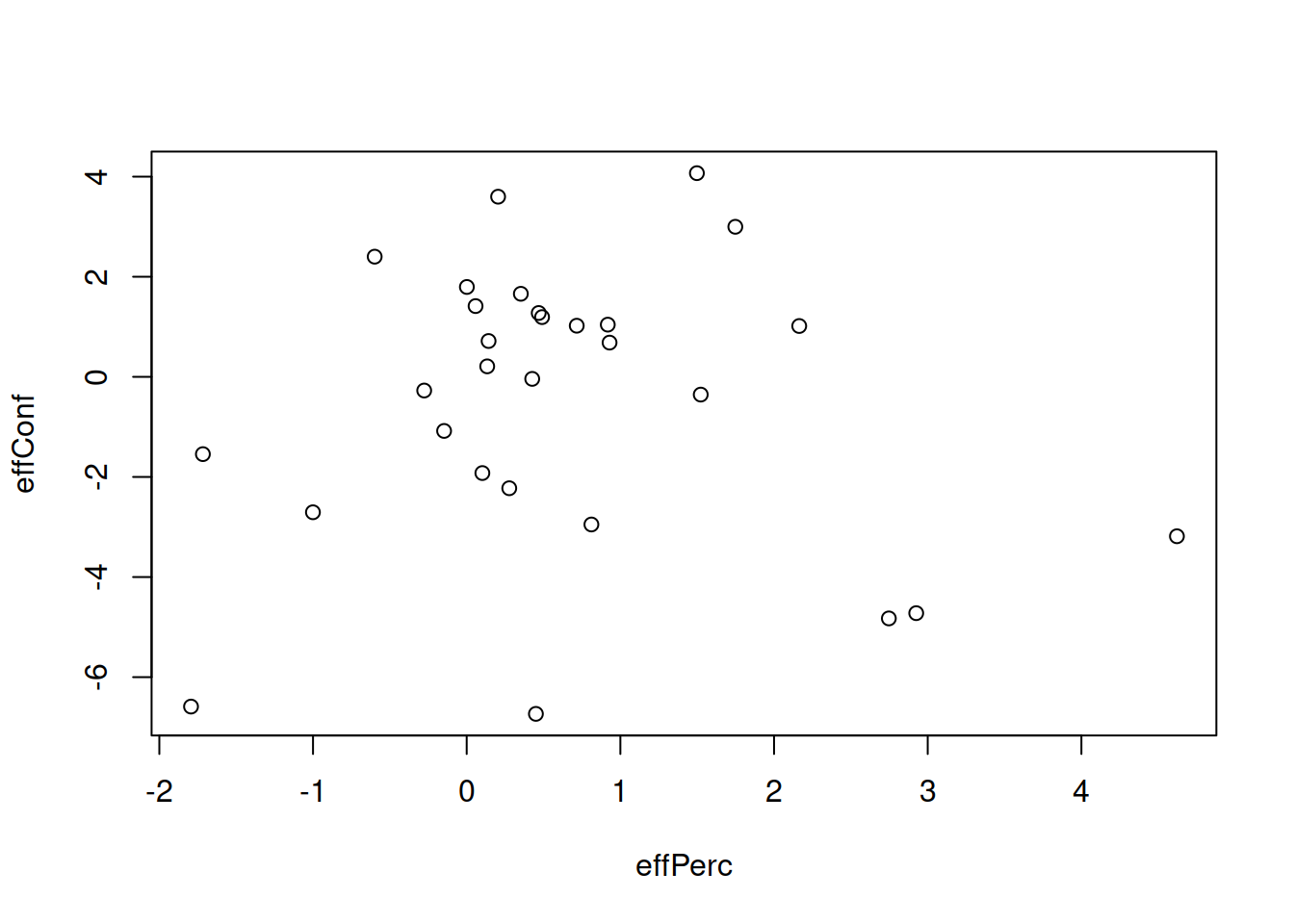


Figure S2. Scatter plot of the stimulus effect on confidence (vertical axis) over the stimulus effect on perceptual performance (horizontal axis) for Experiment 2.

### Within-subjects mediation analysis

For Experiment 1, we defined stimulus, performance, and confidence in the same way as described in the correlation analysis above (stimulus as log(GSNR/LSNR), performance as perceptual d’, and confidence as z(proportion of high-confidence responses)), computed for each of the four levels of LSNR. We then used the bmlm R package to conduct a Bayesian multilevel mediation analysis. Table S1 shows the details of the effect estimates, and Figure S3 shows the path plot of the mediation. The mediation effect (me in Table S1 or a*b in Figure S3) was insignificant (mean estimate = 0.03, standard error = 0.02, 95% credible interval = [-0.02, 0.07]), while the direct effect (cp in Table S1 and c’ in Figure S3) of stimulus on confidence was significant (mean estimate = 0.13, standard error = 0.04, 95% credible interval = [-0.05, 0.23]).

## Parameter Mean SE Median 2.5% 97.5% n_eff Rhat

## 1 a 0.08 0.05 0.08 -0.01 0.17 3185 1.00

## 2 b 0.40 0.12 0.39 0.19 0.66 859 1.01

## 3 cp 0.13 0.04 0.13 0.05 0.23 1461 1.00

## 4 me 0.03 0.02 0.02 -0.02 0.07 2661 1.00

## 5 c 0.16 0.05 0.15 0.06 0.26 1802 1.00

## 6 pme 0.15 0.41 0.15 -0.20 0.47 3316 1.00

Table S1. Results of the within-subjects mediation analysis for Experiment 1. a, b, and cp correspond to the path effects of a, b, and c’ in Figure S3 below. me stands for the mediation effect. c stands for the total effect of stimulus on confidence. pme stands for the percentage of mediation.


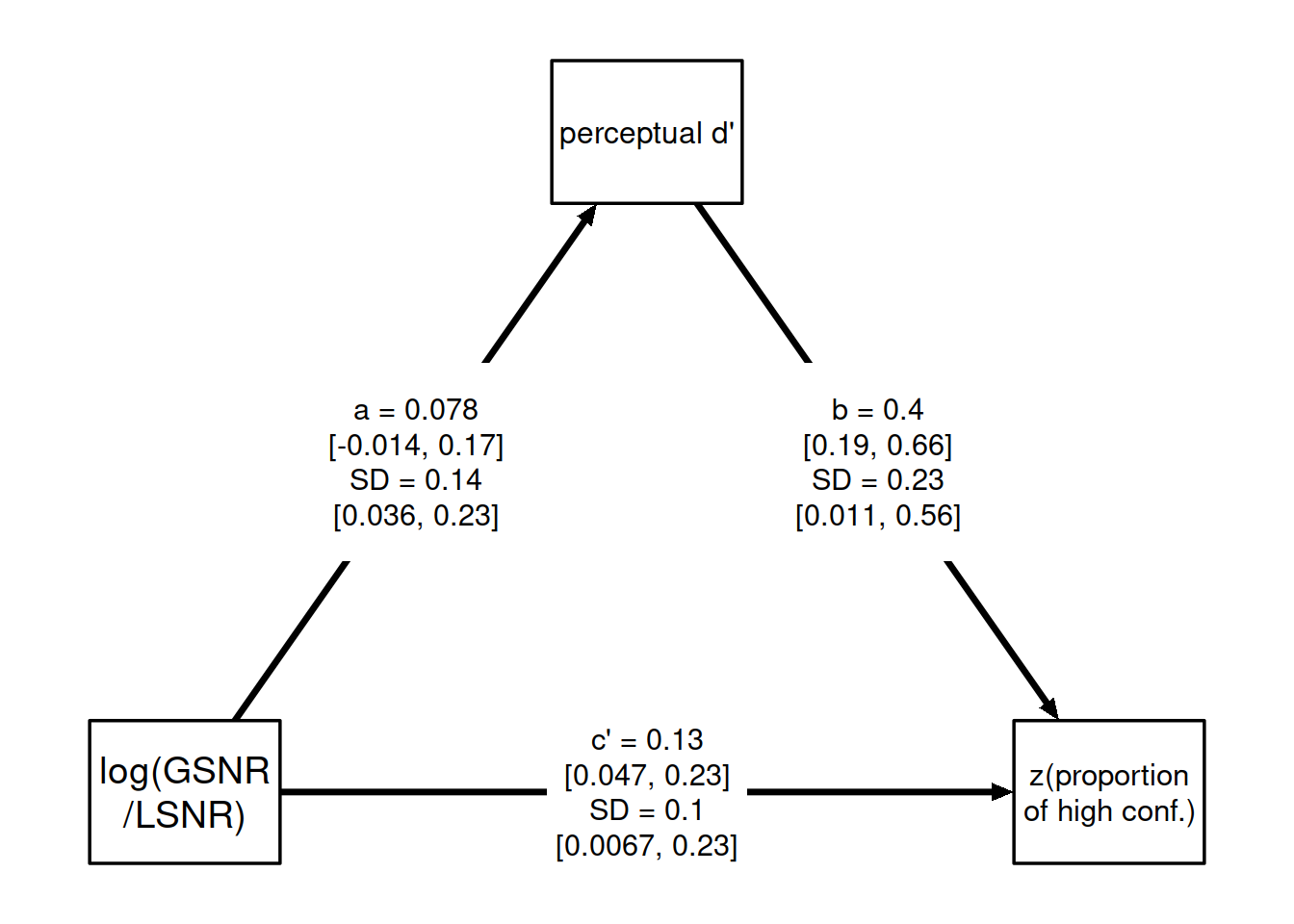


Figure S3. The path plot for the within-subjects mediation analysis of Experiment 1. Paired values in squared brackets indicate 95% credible intervals of the estimated value above them.

For Experiment 2, we defined stimulus, performance, and confidence in the same way as described in the correlation analysis above (stimulus as log(GSNR/LSNR), performance measured inversely as imprecision (sigma), and confidence measured inversely as the average bet span), computed for each of the three levels of LSNR. We used the bmlm R package to conduct a Bayesian multilevel mediation analysis. Table S2 shows the details of the effect estimates, and Figure S4 shows the path plot of the mediation. Both the mediation (indirect) effect via perceptual performance (mean estimate = -5.70, standard error = 2.51, 95% credible interval = [-11.22, -1.51]) and the direct effect of stimulus on confidence were significant (mean estimate = 11.14, standard error = 3.84, 95% credible interval = [-18.78, -3.46]).

## Parameter Mean SE Median 2.5% 97.5% n_eff Rhat

## 1 a -6.40 1.73 -6.40 -9.80 -3.02 1890 1.00

## 2 b 0.71 0.33 0.73 -0.03 1.32 632 1.01

## 3 cp -11.14 3.84 -11.11 -18.78 -3.46 1096 1.00

## 4 me -5.70 2.51 -5.43 -11.22 -1.51 1435 1.00

## 5 c -16.84 3.62 -16.81 -24.19 -9.81 1302 1.00

## 6 pme 0.35 0.16 0.33 0.09 0.71 1378 1.00

Table S2. Results of the within-subjects mediation analysis for Experiment 2. Notations and abbreviations are the same as those in Table S1.


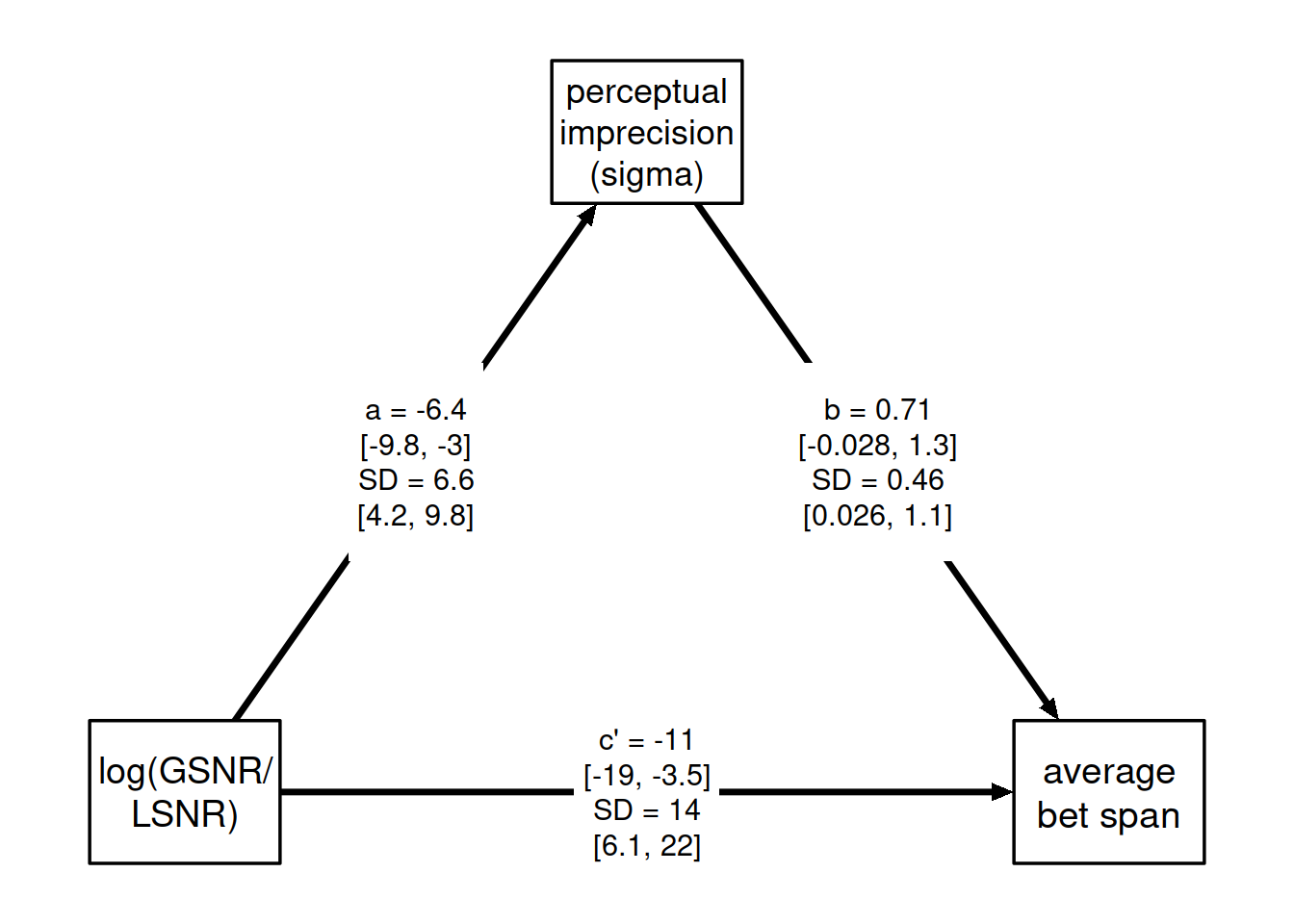


Figure S4. The path plot for the within-subjects mediation analysis of Experiment 2 Paired values in squared brackets indicate 95% credible intervals of the estimated value above them.
